# Fenretinide Improves Intestinal Barrier Function and Mitigates Alcohol Liver Disease

**DOI:** 10.3389/fphar.2021.630557

**Published:** 2021-03-18

**Authors:** Xiao-Han Tang, Marta Melis, Karen Mai, Lorraine J. Gudas, Steven E. Trasino

**Affiliations:** ^1^ Department of Pharmacology, Weill Cornell Medical College of Cornell University, New York, NY, United States; ^2^ Nutrition Program, Hunter College, City University of New York, New York, NY, United States

**Keywords:** alcohol, fenretinide, gut barrier, endotoxemia, toll-like receptor 4, steatosis, inflammation, tumor necrosis factor-α

## Abstract

Alcohol liver disease (ALD) is a major cause of liver-related mortality globally, yet there remains an unmet demand for approved ALD drugs. The pathogenesis of ALD involves perturbations to the intestinal barrier and subsequent translocation of bacterial endotoxin that, acting through toll-like receptor 4 (TLR4), promotes hepatic inflammation and progression of ALD. In the present study we investigated the ability of fenretinide (Fen) [N-(4-hydroxyphenyl) retinamide], a synthetic retinoid with known anti-cancer and anti-inflammatory properties, to modulate intestinal permeability and clinical hallmarks of ALD in a mouse model of chronic ethanol (EtOH) exposure. Our results show that EtOH-treated mice had reductions in mRNA and protein expression of intestinal tight junction proteins, including claudin one and occludin, and increases in intestinal permeability and endotoxemia compared to pair-fed mice. Also, EtOH-treated mice had marked increases in hepatic steatosis, liver injury, and expression of pro-inflammatory mediators, including TNF-α, and TLR4-positive macrophages, Kupffer cells, and hepatocytes in the intestines and liver, respectively. In contrast, EtOH + Fen-treated mice were resistant to the effects of EtOH on promoting intestinal permeability and had higher intestinal protein levels of claudin one and occludin. Also, EtOH + Fen-treated mice had significantly lower plasma levels of endotoxin, and reductions in expression of TNF-α and TLR4 positive macrophages, Kupffer cells, and hepatocytes in the intestine and liver. Lastly, we found that EtOH + Fen-treated mice exhibited major reductions in hepatic triglycerides, steatosis, and liver injury compared to EtOH-treated mice. Our findings are the first to demonstrate that Fen possesses anti-ALD properties, potentially through modulation of the intestinal barrier function, endotoxemia, and TLR4-mediated inflammation. These data warrant further pre-clinical investigations of Fen as a potential anti-ALD drug.

## Introduction

Alcohol liver disease (ALD) is responsible for almost half of all liver-related deaths around the world and is the second most common cause of all liver transplants (Rehm et al., 2013). There are currently no FDA-approved drugs for the treatment of ALD, and therapeutic drugs that aid in alcohol abstinence are generally ineffective (Osna et al., 2017). The pathogenesis of ALD is complex and multifactorial, but multiple lines of evidence support that chronic alcohol intake disrupts gastrointestinal tight junction proteins (TJPs), increasing the gut translocation of bacterial lipopolysaccharide (LPS) into the systemic blood, where it stimulates the release of pro-inflammatory mediators including tumor necrosis factor-α (TNF-α) from liver Kupffer cells through toll-like receptor 4 (TLR4) (Bode et al., 1987; Rao, 2009; Samuelson et al., 2019). Moreover alcohol sensitizes Kupffer cells and hepatocytes to the pro-inflammatory effects of LPS and TNF-α, creating a vicious gut-liver inflammation cycle (Petrasek et al., 2010).

Conversely, mice harboring inactivating mutations in TLR4 or TNF- α receptor I (TNFRI) are protected against alcohol-mediated gut barrier disruption, hepatic steatosis, and progression ALD (Uesugi et al., 2001; Chen et al., 2015). Moreover, a convincing body of evidence shows that anti-inflammatory dietary medium chain fatty acids and ω-3 polyunsaturated fatty acids (PUFAs) preserve gut barrier and inhibit TLR4 mediated inflammation and ALD in alcohol-fed rodents (Kirpich et al., 2012; Zhong et al., 2013). Despite the known role that dysregulation of the gut barrier has in the development of ALD (Bode et al., 1987; Rao, 2009; Samuelson et al., 2019), to date no drugs have been examined for their potential to protect barrier function and mitigate the progression of hallmarks of ALD, including hepatic steatosis, steatohepatitis, and fibrosis.

Fenretinide (Fen) [N-4-hydroxyphenyl-retinamide, 4-HPR] is a synthetic derivative of all-trans retinoic acid that has been primarily studied for its promising anti-cancer properties in numerous clinical trials of adult and pediatric cancers (Veronesi et al., 2006; Cooper et al., 2017). Fen inhibits cancer cell proliferation through the induction of reactive oxygen species (ROS) and apoptosis through mechanisms that are incompletely understood [reviewed in (Hail et al., 2006)]. Recently, studies have demonstrated that Fen possesses anti-inflammatory properties (López-Vales et al., 2010; Lachance et al., 2013; Kanagaratham et al., 2014; Lin et al., 2016), marked by inhibition of LPS-induced expression of inflammatory mediators such as IL-1β and TNF-α in mouse models of allergic asthma (Kanagaratham et al., 2014), and LPS-disruption of the blood brain barrier TJP occludin (Li et al., 2020). Moreover, evidence shows that Fen also possesses anti-diabetic properties (Preitner et al., 2009) and can mitigate hepatic fatty steatosis in genetic and dietary models of non-alcoholic fatty liver disease (NAFLD) (Preitner et al., 2009; Koh et al., 2012), which shares pathological features of ALD (Sanyal et al., 2016). Therefore, in light of these data, and its history of safety and low toxicity in humans (Veronesi et al., 2006; Cooper et al., 2017), we sought to examine for the first time the effects of Fen on intestinal barrier function and progression of ALD in a model of chronic ethanol exposure in mice.

## Materials and Methods

### Animals

All animal experiments were conducted in accordance with the Guide for the Care and Use of Laboratory Animals as adopted and promulgated by the U.S. National Institutes of Health and the Institutional Animal Care and Use Committee (IACUC) guidelines at Hunter College.

### Liquid Ethanol Diet

Male Wild type (Wt) C57BL/6J mice (8–9-week-old) (*n* = 20) were purchased from Jackson Laboratory and were housed in standard cages with 2-mice per cage and fed standard laboratory chow (rodent diet #2014, Harlan-Teklad, Madison, WI, United States) prior to initiation of liquid diet feeding. After 7 days, mice were switched from chow diet to a nutritionally sufficient, control liquid diet (Lieber-DeCarli-shake and pour control liquid diet, Bio-Serve, Inc. diet #F1259SP) for 5 days. After 5 days, mice were randomly assigned to either remain on a liquid control diet (*n* = 8) or switched to an ethanol liquid diet (*n* = 12) (Lieber-DeCarli-shake and pour ethanol liquid diet, Bio-Serve, #F1258SP) for acclimatization as follows: 1% ethanol (v/v) for 2 days, 2% (v/v) for 3 days. After 5 days, ethanol-fed mice were switched to either 5% ethanol v/v (approximately 27.6% kcal from ethanol) plus vehicle [0.1% DMSO] in their liquid diet (EtOH-treated, *n* = 6), or 5% ethanol plus fenretinide (Apexbio, Inc) [10 mg/kg/day] (EtOH + Fen). A group of liquid control-fed mice (*n* = 4) were switched to Fen at a dose of 10 mg/kg/day. All control diet-fed mice were pair-fed (no EtOH) to the mean 24 h intake of 5% EtOH-treated mice. Control and ethanol diets were prepared fresh daily. All groups remained on their diets for 25 days. Changes to food intake and body weight were similar across all experimental groups throughout the study period (Supplementary Figures S1A,B).

### *In vivo* Intestinal Permeability Assay

Determination of intestinal permeability was performed as previously described (Volynets et al., 2016). Mice were fasted for 4 h and then administered fluorescein isothiocyanate (FITC)-dextran D4000 (4-kDa, dissolved in sterile PBS 100 mg/ml) (Sigma-Aldrich, #FD4) at a dose of 600 mg/kg body weight by oral gavage. Two hours after gavage, blood was collected from the tail vein in heparinized tubes, protected from light, and immediately centrifuged (2,000 ×*g*) to collect plasma fractions. Plasma FITC-dextran was measured at an excitation wavelength of 490 nm and an emission wavelength of 520 nm on a fluorescence spectrophotometer. Standard curves were generated with serial dilutions of FITC-dextran in nontreated mouse plasma to determine plasma concentrations.

### Plasma Biochemical Assays

Blood samples for the following biochemical assays were collected from the tail vein 2 h after lights off when blood alcohol content (BAC) levels peak in rodents (Freund, 1970). BAC levels were measured in plasma samples using a commercially available ethanol assay kit (Sigma-Aldrich, Inc.). Plasma alanine aminotransferase (ALT) and aspartate aminotransferase (AST) activity were measured using commercially available enzymatic assay kits (BioAssay Systems, Inc.) following the manufacturer’s protocol. Plasma endotoxin (LPS) was measured under pyrogen-free conditions using a Limulus amebocyte lysate (LAL) chromogenic LPS kit following the manufacturer’s protocol (Thermo, Inc).

### Immunohistochemistry

Immunohistochemistry (IHC) was performed as previously described (Trasino et al., 2016). Briefly, after deparaffinization and re-hydration with graded alcohol concentrations, deparaffinized ileum and liver tissue sections were incubated with the following primary antibodies overnight at 4°C: Occludin 1 (Rabbit polyclonal, #13409, Proteintech, Inc.), claudin 1 (Rabbit polyclonal, #13050, Proteintech, Inc.), TNF-α (Rabbit polyclonal, #A0277, Abclonal, Inc.), TLR4 (Mouse monoclonal, clone 76B357.1, Novus, Inc.), F4/80 (Rat monoclonal, #ab6640, Abcam, Inc.), α-Smooth Muscle Actin (Rabbit monoclonal #19245, Cell Signaling Inc.), and 4 Hydroxynonenal (4-HNE) (Rabbit polyclonal, #ab46545, Abcam, Inc.). After overnight incubation, samples were treated with the appropriate HRP- or fluorescent-conjugated secondary antibodies (SuperBoost Goat anti-Rabbit Poly HRP, Thermo, Inc.), or goat anti-rabbit IgG, Alexa Fluor 488 and 594 (Thermo, Inc.). HRP-conjugated antibodies were visualized with 3,3-diaminobenzidine (DAB).

### Liver Fibrosis and Collagen Deposition

To determine liver collagen deposition and fibrosis, paraffin embedded liver sections were stained with Masson’s Trichrome Kit (Poly Scientific, Bayshore, NY, United States), according to the manufacturers’ protocol. Liver collagen was then analyzed by color densitometry analysis using Fiji ImageJ software as described (Schindelin et al., 2012).

### Quantitation of Immunofluorescence and Immunohistochemistry

For quantitation of intestinal and liver antibody staining, slide images were photographed using a Nikon Ts2-inverted fluorescent microscope. Approximately 10-15 FITC-488, TRITC-594, and DAB tissue positive fields per slide, with one slide per mouse, and four to six mice per dietary group, for a total of 40–90 antibody positive fields per experimental group, were analyzed by color densitometry analysis using Fiji ImageJ software as described (Schindelin et al., 2012).

### RNA Isolation and Quantitative RT-PCR

Total RNA was extracted and purified from 2 cm portions of ileum (approximately 10–15 mg) and liver samples (approximately 10–15 mg) using the RNeasy Mini Kit (Qiagen, Inc.). RNA concentration and purity were measured on a Nanodrop One (Thermo, Inc.). Total RNA (1 μg) was used to synthesize cDNA using the RevertAid First Strand cDNA Synthesis Kit (Thermo, Inc.). Quantitative RT-PCR (q-PCR) was performed as previously described (Trasino et al., 2016) using SYBR Green PCR master mix on an Agilent Mx3000P Real Time PCR system (Agilent, Inc.). Gene specific primers (Supplementary Table S1) were used to amplify target mRNAs that were normalized to the internal control mRNA, *36B4*. Relative gene expression fold changes were calculated using the delta, delta Ct method (Livak and Schmittgen, 2001).

### Quantification of Intestinal Bacterial

Total DNA was extracted and purified from the intestinal cecum using QIAamp DNA Mini Kit (Qiagen, Inc). Cecum DNA (1 μg) was amplified for total bacterial species using universal bacterial primers (Horz et al., 2005) (Supplementary Table S1) on an Agilent Mx3000P Real Time PCR system (Agilent, Inc.). The relative levels of total bacterial species are expressed as relative fold change compared to pair-fed mice.

### Statistical Analysis

Group means differences are reported as means +/− SD and were analyzed using repeated measures ANOVA and Dunnett’s multiple comparison test. Group means were treated as independent variables and computed using ANOVA and Dunnett’s multiple comparison test when sample numbers were not equal. Significant differences were defined as *p*-values of less than 0.05, and all usage of the term “significant” throughout the text refers to means differences with a *p* < 0.05. All statistical analyses were performed using GraphPad Prism version 8.0 statistical software (GraphPad Software, Inc.).

## Results

### Fenretininde Reduces Ethanol-Induced Intestinal Permeability and Endotoxemia

**FIGURE 1:**
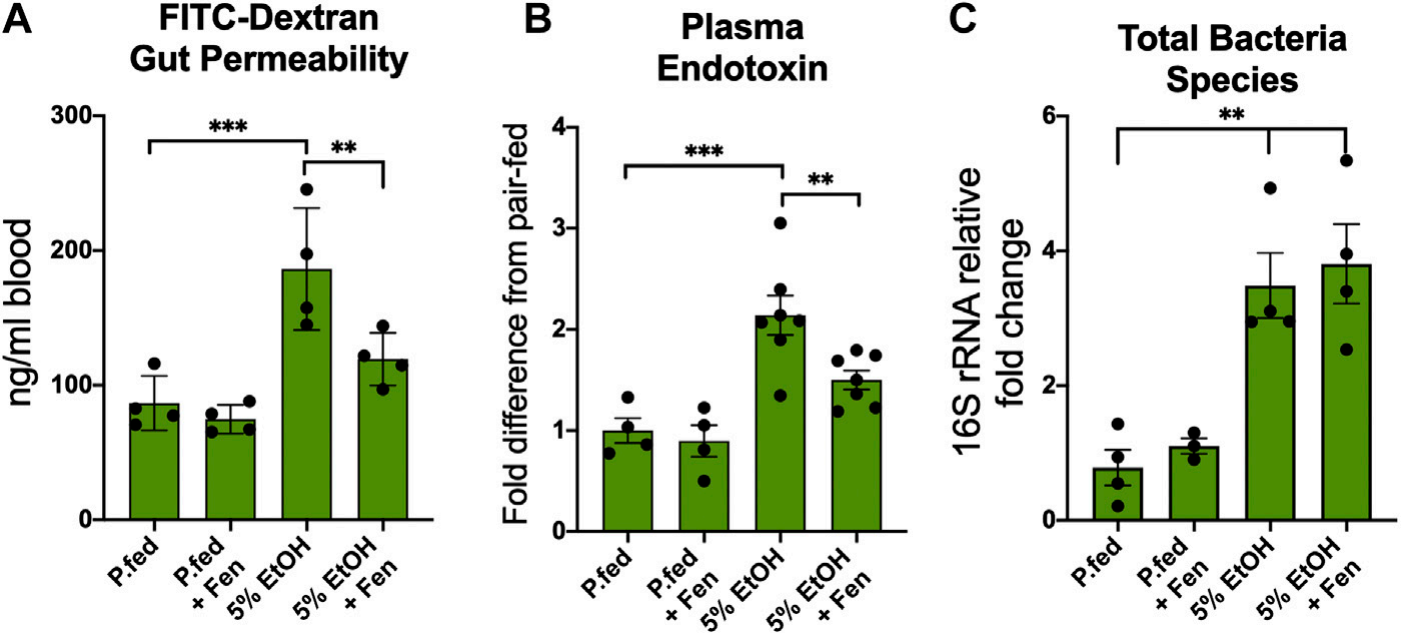
Fenretininde prevents intestinal hyper-permeability and endotoxemia. **(A)** Blood levels of 4-kDa fluorescein isothiocyanate (FITC) dextran 2 h after oral gavage. **(B)** Plasma endotoxin (LPS) fold change 2 h after peak blood alcohol content relative to pair-fed mice. **(C)** Intestinal total bacterial species measured as cecum 16S rDNA fold change relative to pair-fed mice. All data error bars represent ±SD, with **p < 0.01, ***p < 0.001.

Alcohol-induced intestinal hyper-permeability and endotoxemia are key contributors to the pathogenesis of ALD (Bode et al., 1987; Rao, 2009); therefore, we measured intestinal permeability *in vivo* with an oral dose FITC-dextran into the systemic blood as previously described (Volynets et al., 2016). We found that chronic alcohol exposure markedly altered intestinal permeability, as we detected over a 110% increase in blood FITC-dextran concentrations in EtOH-treated compared to pair-fed mice (Figure 1A), whereas blood concentrations of FITC-dextran levels were reduced by 36% in EtOH + Fen-treated compared to EtOH-treated mice (Figure 1A). No differences in FITC-dextran blood levels were detected between pair-fed and pair-fed + Fen-treated mice (Figure 1A). Given that the intestine is the source of bacterial LPS in the pathogenesis of ALD (Keshavarzian et al., 2009), we next sought to determine the effect of Fen treatments on LPS translocations into the systemic blood in both EtOH cohorts of mice. We found that systemic LPS levels in EtOH-treated mice were increased ∼2.2-fold compared to pair-fed mice and, consistent with the reductions in gut permeability (Figure 1A), EtOH + Fen-treated mice showed reductions in systemic LPS and endotoxemia of over 30% compared to EtOH-treated mice (Figure 1B).

Human and experimental animal models have demonstrated that chronic alcohol exposure is associated with intestinal microbiota dysbiosis, which is marked by bacterial overgrowth that can contribute to increased intestinal hyper-permeability (Rao, 2009; Yan et al., 2011). Therefore, we next measured intestinal bacterial load in the cecum by qPCR using universal 16s rRNA bacterial primers (Supplementary Table S1). Consistent with previous rodent studies (Keshavarzian et al., 2009; Yan et al., 2011), we found that the cecal total bacteria contents in EtOH-treated were approximately 4.5-fold higher compared to pair-fed mice (Figure 1C). We did not detect mean differences in cecal total bacterial content between EtOH-treated and EtOH + Fen-treated mice (Figure 1C). Given that food intake and peak blood alcohol levels were unchanged between EtOH-treated and EtOH + Fen-treated mice (Supplementary Figures S1A,C), the Fen-associated improvements to the intestinal barrier and systemic endotoxemia were likely not a result of alterations in gut bacterial overgrowth or alcohol exposure.

### Fenretininde Increases Expression of Intestinal Tight Junction Proteins

**FIGURE 2:**
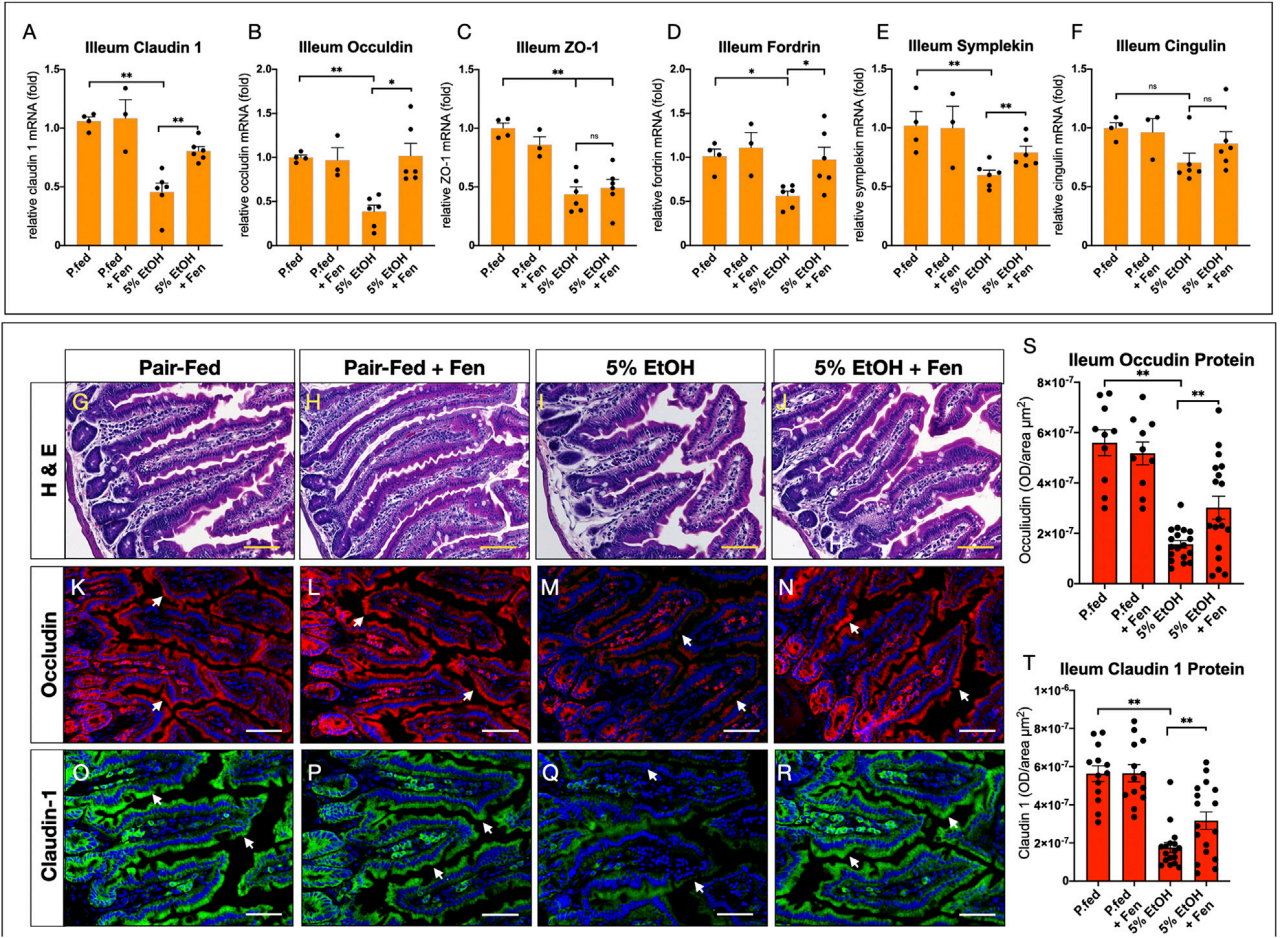
Fenretininde increases intestinal expression of tight junction proteins. Ileum mRNA levels of tight junction proteins **(A)** *Claudin 1,* **(B)**, *Occludin,* **(C)** *Z O -1,* **(D)** *Fordrin,* **(E)** *Symplekin,* **(F)** *Cingulin* were measured using qPCR as described in materials and methods. **(G–J)** Representative images of hematoxylin and eosin stain ileum sections. Magnification: ×100; Scale Bar = 50 μm. Representative immunofluorescence (IF) images of ileum sections stained with antibodies against: occludin K-N), or claudin one O-R). Magnification: ×100; Scale Bar = 50 μm. Quantification of ileum IF optical intensity of occludin S), and claudin 1 T). All data errors bars represent ±SD, with **p < 0.01, ns = not significant.

Intestinal barrier function is mediated by tight-junction proteins (TJPs), such as occludin, claudins, zona occludins 1 (ZO-1), and other adaptor proteins such as cingulin and fordrin (Groschwitz and Hogan, 2009). Numerous lines of evidence demonstrate that alcohol-mediated increases in intestinal permeability are associated with perturbations and reductions in the expression and function of TJPs, such as occludin and claudins, permitting paracellular translocation and translocation of bacterial products and further exacerbating local and systemic endotoxemia and inflammation (Wang et al., 2014; Samuelson et al., 2019). Therefore, given that we detected increases in gut permeability and endotoxemia in EtOH-treated mice, we assessed mRNA and protein levels of TJPs in the intestinal ileum using qPCR and immunofluorescence (IF). By qPCR analysis EtOH-treated mice showed approximately 2.5-fold reductions in ileum mRNA levels of TJPs claudin 1, occludin, and ZO-1 (Figures 2A–C). We also found that, compared to pair-fed mice, ileum mRNA levels of the TJP adaptor proteins fordin and symplekin were reduced in EtOH-treated mice by 1.8 and 1.7-fold, respectively (Figures 2D,E). Ileum mRNA levels of the adaptor protein cingulin were reduced in EtOH-treated mice by approximately 1.4-fold, but these reductions did not reach statistical significance (Figure 2F). In contrast, ileum mRNA transcripts of the TJPs claudin 1, occludin, fordin, and symplekin were significantly higher in EtOH + Fen-treated compared to EtOH-treated mice (Figures 2A,B,D,E). We did not detect statistically significant differences in ileum mRNA levels of ZO-1 (Figure 2C) and cingulin (Figure 2F) between EtOH-treated and EtOH + Fen-treated mice. Ileum mRNA levels of all of the TJPs measured were unchanged between pair-fed and pair-fed + Fen-treated mice (Figures 2A–F). Using IF, we found that ileum immunoreactivity for the TJPs occludin (Figures 2M *vs*. 2K, white arrows, 2S) and claudin 1 (Figure 2Q *vs*. 2O, white arrows, 2T), was reduced in EtOH-treated mice by 72 and 68%, respectively, compared to pair-fed mice. In contrast and consistent with our qPCR findings, ileum immunoreactivity of occludin (Figure 2N *vs*. 2M, white arrows, 2S) and claudin 1 (Figure 2R *vs*. 2Q, white arrows, 2T) was increased by 93 and 80%, respectively, in EtOH + Fen-treated mice compared to EtOH-treated mice. Despite the reductions in ileum TJPs, ileum histological analysis did not show any gross morphological differences in intestinal villi across all experimental groups (Figures 2G–J).

### Fenretininde Reduces Intestinal Toll-like Receptor 4-Positive Macrophages and Inflammatory Mediators

Toll-like receptor 4 (TLR4) is a key component of the innate immune response that binds to bacterial LPS, triggering an anti-microbial response that includes secretion of inflammatory cytokines, including TNF-α (Uematsu and Akera, 2006). TLR4 is expressed in cells of the innate immune response, including macrophages (Uematsu and Akira, 2006), and a convincing body of evidence shows that TLR4 and LPS mediate reductions in intestinal barrier function and expression of TJPs (Guo et al., 2015; Li et al., 2013; Uesugi et al., 2001). Given our findings of increased intestine hyperpermeability and LPS translocation in EtOH-treated cohorts (Figures 1A,B), we next examined ileum expression of TLR4 using qPCR and immunofluorescence (IF). Our qPCR data show that compared to pair-fed, EtOH-treated mice exhibited an approximately 3-fold increase in ileum TLR4 transcripts (Supplementary Figure S2A). EtOH + Fen-treated mice displayed approximately 37% reductions in ileum TLR4 mRNA levels compared to the EtOH-treated mice (Supplementary Figure S2A). Compared to pair-fed, we did not detect changes to TLR4 mRNA in pair-fed + Fen-treated mice (Supplementary Figure S2A).

We next sought to determine ileum TLR4 protein levels, and more specifically, the percentage of TLR4 positive macrophages in the ileum lamina in pair-fed and EtOH-treated groups. Using double IF and antibodies against the macrophage glycoprotein F4/80 and TLR4, we detected, compared to pair-fed mice, 3-fold and 2-fold increases in villi lamina F4/80 positive macrophages in EtOH-treated and EtOH + Fen-treated mice, respectively (Supplementary Figures 2B,G *vs*. 2F, and 2I *vs*. 2F). The differences in the percentages of villi lamina F4/80 positive macrophages between EtOH-treated and EtOH + Fen-treated mice did not meet statistical significance (Supplementary Figure S2B). However, we found that, compared to pair-fed mice, the percentages of F4/80:TLR4 double positive macrophages increased by 13-fold in EtOH-treated mice (Supplementary Figures S2B,G *vs*. S2F, [white arrows]), and were reduced by 46% in EtOH-Fen mice (Supplementary Figures S2B,I *vs*. S2G [white arrows]). Pair-fed-Fen mice, compared to paid-fed mice, showed no differences in the percentages of villi lamina F4/80 positive and F4/80:TLR4 double positive macrophages (Supplementary Figures S2B,H *vs*. S2F).

We next measured ileum mRNA levels of TNF-α, a major downstream cytokine of TLR4 (Petrasek et al., 2010; Uematsu and Akira, 2006), and of the TNF-α receptor (TNFR1), both of which play key roles in alcohol-mediated disruption of the intestinal barrier function and liver injury in ALD (Chen et al., 2015; Yin et al., 1999). Our qPCR data show that ileum TNF-α mRNA levels were increased by ∼2.3-fold in EtOH-treated compared to pair-fed mice (Supplementary Figure S2C), whereas ileum TNFR1 transcripts were increased by approximately 1.5-fold in EtOH-treated *vs*. pair-fed mice, but these changes were not statistically significant (Supplementary Figure S2D). Using IHC, we sought to confirm our qPCR findings and found, similarly, that ileum TNF-α immunoreactivity was increased by 2.2-fold in EtOH-treated compared to pair-fed mice (Supplementary Figures S2E,K *vs*. S2J). These data are consistent with a previous study showing that chronic alcohol intakes markedly increase intestinal TNF-α mRNA and protein levels in mice and humans (Chen et al., 2015). Our qPCR and IHC analysis also showed that, compared to EtOH-treated mice, ileum mRNA and immunoreactivity levels of TNF-α were reduced in EtOH + Fen-treated mice by 44% and 39%, respectively (Supplementary Figures S2C,M *vs*. S2K,E). We found no differences in ileum mRNA levels of TLR4, TNF-α, and TNF-α protein between pair-fed and pair-fed-Fen-treated mice (Supplementary Figures S2A,C,E), and TNFR1 mRNA levels between EtOH-treated and EtOH + Fen-treated mice (Supplementary Figure S2D).

### Fenretinide Does Not Alter Tight Junction Protein Expression Through Modulation of Oxidative Stress

Previous studies have demonstrated that Fen can inhibit LPS and TNF-α mediated inflammation (López-Vales et al., 2010), and LPS mediated disruption of TJPs at the blood brain barrier (BBB) though activation of the Nrf2-antioxidant response element signaling pathway and reductions in reactive oxygen species (ROS) and oxidative stress (Li et al., 2020). Moreover, data show that modulation of cellular ROS is involved in the chemopreventive actions of Fen in a number of cancers (Hail et al., 2006; Sheikh et al., 1995). Therefore, using IHC, we next measured ileum levels of 4-hydroxynonenal (4-HNE), a lipid peroxidation product and a marker of oxidative stress (Esterbauer et al., 1991). We found that, compared to paid-fed mice, EtOH-treated and EtOH + Fen-treated mice showed an almost 10-fold increase in ileum levels of 4-HNE (Supplemental Figures S3B,D *vs*. S3A,E), suggesting a large increase intestinal ROS and oxidative stress. We found no differences in the ileum levels of 4-HNE between EtOH-treated and EtOH + Fen-treated mice, however (Supplementary Figures S3D *vs*. S3B,E). Using qPCR we next measured the mRNA transcripts of Gsta1 and Gstm1, two Nrf-regulated genes involved in the cellular antioxidant defense response (Ma, 2013), and found, consistent with our 4-HNE IHC data showing increased levels of ileum oxidative stress, that EtOH-treated mice had 2.4 and 3.2-fold increases in ileum transcripts of the Nrf-target genes Gsta1 and Gstm1, respectively (Supplemental Figures S3F,G). Also consistent with our 4-HNE-IHC data, we did not detect differences in ileum mRNA levels of Gsta1 and Gstm1 between EtOH-treated and EtOH + Fen-treated mice (Supplemental Figures S3F,G).

### Fenretininde Reduces Hepatic Steatosis and Liver Injury

**FIGURE 3:**
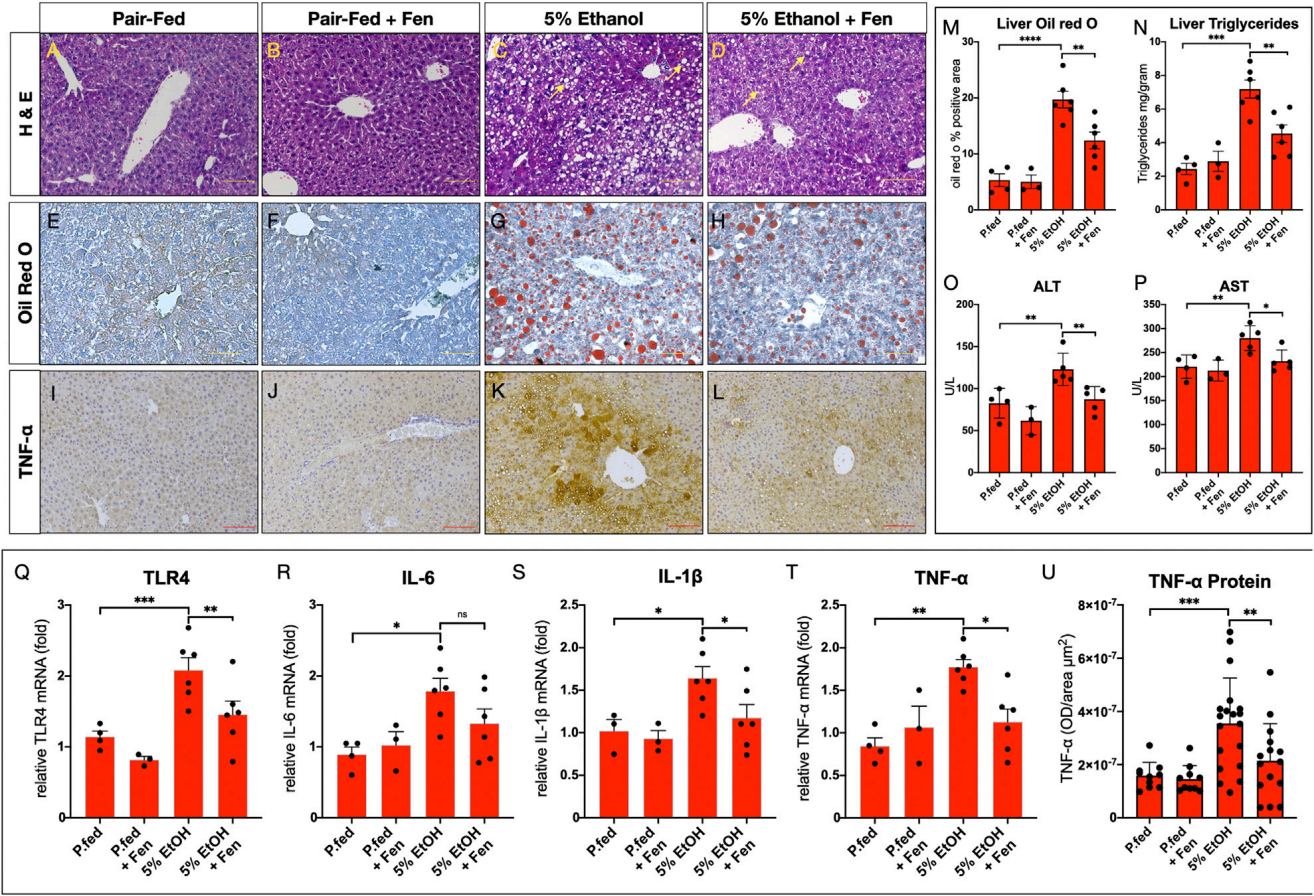
Fenretininde reduces alcohol liver disease (ALD) and liver inflammation. **(A–D)** Representative images of hematoxylin and eosin; **(E–H)** Oil-Red O-stained liver sections Magnification: ×100; Scale Bar = 50 μm; **(I–L)** Representative immunohistochemistry (IHC) images of liver sections stained with an antibody against tumor necrosis factor α (TNF-α) Magnification: ×100; Scale Bar = 50 μm. **(M)** Quantification of liver Oil-red-O stained liver sections. **(N)** Liver triglyceride concentrations (mg/Gram tissue weight). Plasma activity levels (U/L) of **(O)** alanine amino transferase (ALT) and **(P)** aspartate amino transferase (AST). Hepatic mRNA levels of inflammatory mediators **(Q)** *TLR4*, **(R)** *IL-6*, **(S)** *IL-1β*, **(T)** *TNF-α*. **(U)** Quantification of hepatic IHC for TNF-α protein levels. All data errors bars represent ±SD, with *p < 0.05, **p < 0.01. ***p < 0.001, ns = not significant.

We next evaluated the effects of ethanol feeding on liver steatosis and injury in EtOH-treated and EtOH + Fen-treated mice. Histological evaluations of livers showed that compared to pair-fed mice, EtOH-treated mice developed hepatic steatosis (Figures 3C *vs*. 3A [yellow arrows]) marked by 4-fold and 3-fold increases in Oil-red O lipid staining (Figures 3G *vs*. 3E,M) and hepatic triglyceride levels (Figure 3N), respectively. In contrast, we found that livers of EtOH + Fen-treated mice showed marked reductions in hepatic steatosis compared to EtOH-treated mice (Figures 3D *vs*. 3C[yellow arrows]), with reductions in Oil-red O lipid staining (Figures 3H *vs*. 3G,M) and hepatic triglyceride levels (Figure 3N) of 34% and 39%, respectively. Given these modulations of liver triglycerides and steatosis, we next measured hepatic mRNA levels of key lipogenic mediators involved in ALD-steatosis, including FASN, SREBP1-c, ACC1 and PPARγ, by qPCR. Our data show that, compared to pair-fed mice, hepatic mRNA levels of both *FASN* and *PPAR*γ were elevated by 2–2.5-fold (Supplementary Figures S4A,B), in EtOH-treated and ETOH + Fen-treated mice. Hepatic *FASN* transcripts were 20% lower in EtOH + Fen-compared EtOH-treated mice (Supplementary Figure S4A); however, we found no significant differences in hepatic mRNA levels of *FASN* or *PPARγ* between the EtOH-treated and EtOH + Fen-treated groups (Supplementary Figures S4A,B). Also, hepatic transcripts of *SREBP1c* and *ACC1* were not significantly different among all groups (Supplementary Figures S4C,D).

Alcohol-mediated liver injury can be assessed by plasma levels of the hepatic transaminases alanine aminotransferase (ALT) and aspartate aminotransferase (AST) (Reichling and Kaplan, 1988). We found that, compared to pair-fed mice, plasma ALT and AST enzyme activity levels were elevated in EtOH-treated mice by 50% and 27%, respectively (Figure 3O,P). In contrast, plasma levels of ALT and AST were reduced by 30% and 17% in EtOH-Fen-treated compared to EtOH-treated mice (Figures 3O,P). Compared to pair-fed mice, hepatic steatosis and plasma levels of ALT and AST in pair-fed-Fen treated mice were not different (Figures 3M–P).

### Fenretininde Reduces Hepatic Expression of Toll-like Receptor 4 and Inflammatory Mediators

In chronic alcohol exposure and endotoxemia, LPS promotes an inflammatory response through TLR4 (Petrasek et al., 2010), which in the liver is expressed in both hepatocytes (Liu et al., 2002; Jia et al., 2018) and Kupffer cells (Petrasek et al., 2010). Therefore, we next measured hepatic mRNA levels of TLR4 and some of its down-stream inflammatory mediators shown to be involved in alcohol and LPS-induced liver inflammation (Petrasek et al., 2010; Neuman et al., 2015). Our qPCR analysis showed that, compared to pair-fed mice, hepatic transcripts of *TLR4*, *IL-6*, *IL-1β* and *TNF-α* were all significantly increased in EtOH-treated mice (Figures 3Q–T) and, with the exception of *IL-6*, our analysis also showed that hepatic mRNA levels of these inflammatory mediators were significantly lower in EtOH + Fen-treated compared to EtOH-treated mice (Figures 3Q–T). We next used IHC to measure hepatic TNF-α protein levels, given our *TNF-α* qPCR data (Figure 3T) and the key role of TNF-α in the pathogenesis of ALD (Yin et al., 1999; Neuman et al., 2015). We found, compared to pair-fed mice, that hepatic TNF-α protein immunoreactivity and levels increased by more than 120% in EtOH-treated mice (Figures 3K *vs*. 3I,U), and consistent with our qPCR data, that hepatic TNF-α protein levels were reduced by approximately 40% in EtOH + Fen-treated compared to EtOH-treated mice (Figures 3L *vs*.3K,U). Using IF, we next measured hepatic TLR4 protein expression in hepatocytes and Kupffer cells, and found, compared to pair-fed mice, that the percentages of F4/80:TLR4 double positive Kupffer cells (Supplementary Figures S5B *vs*. S5A[white arrows], S5E), and TLR4 positive hepatocytes (Supplementary Figures S5B *vs*. 5A,E) increased by 15.6-fold and 13.7-fold respectively, in EtOH-treated mice. In contrast, compared to EtOH-treated mice, the percentages of F4/80:TLR4 double positive hepatic Kupffer cells (Supplementary Figures 5D *vs*. 5B [white arrows], 5E), and TLR4 positive hepatocytes (Supplementary Figures 5D *vs*. 5B,E), were reduced by 55% and by 60% in EtOH + Fen-treated mice, respectively.

Hepatic steatosis and inflammation can lead to excessive liver collagen deposition, fibrotic scarring, and injury (Osna et al., 2017). Therefore, given that we detected increases in hepatic steatosis (Figures 3M,N) and expression of inflammatory cytokines, including TNF-α (Figures 3Q–U), in the EtOH-cohorts, we next assessed liver fibrosis by using both IHC for the fibrogenic marker α-smooth muscle actin (α-SMA) and Masson’s trichrome staining for collagen. Compared to pair-fed mice, we detected no increases in hepatic levels of α-SMA (Supplementary Figures 4I,J *vs*. 4G,O), or evidence of excessive liver collagen deposition or fibrosis in the EtOH-treated and EtOH + Fen-treated mice (Supplementary Figure 4M,N *vs*. 4K,P).

## Discussion

Multiple lines of evidence indicate that chronic alcohol intake promotes disruption of intestinal barrier integrity (Rao, 2009; Samuelson et al., 2019). The mechanisms through which this occurs are incompletely understood, but modulation of gut bacterial growth (Bode et al., 1987; Rao, 2009; Samuelson et al., 2019), mucosal immunity, and intestinal inflammation have been proposed (Bishehsari et al., 2017). Still, experimental and human data consistently show that in the early stages of ALD, ethanol negatively affects expression of intestinal TJPs (Bode et al., 1987; Rao, 2009; Samuelson et al., 2019), leading to endotoxemia and a pro-inflammatory cascade that damages both the gut and liver (Bode et al., 1987; Rao, 2009; Samuelson et al., 2019).

### Fenretinide Preserves Gut Barrier in Alcohol Liver Disease

Here we demonstrate for the first time that Fen, a synthetic analog of all-trans retinol widely studied for its anti-cancer properties (Veronesi et al., 2006), can mitigate ethanol-induced gut hyper-permeability (Figure 1A), endotoxemia (Figure 1B), and reductions to intestinal mRNA and protein expression of TJPs (Figures 2A–E,S,T). Fen does not directly modulate expression of TJPs, as we did not detect changes in TJP mRNA and protein expression in pair-fed Fen-treated mice (Figures 2A–F,S,T). Moreover, in contrast to findings reported by Li et al. (Li et al., 2020), who showed that Fen mitigates LPS disruption of TJPs at the blood brain barrier through stimulation of the Nrf2-antioxidant pathway, our data show that perseveration of TJP expression in EtOH + Fen-treated mice (Supplementary Figures 2S,T) does not involve modulation of Nrf2 and oxidative stress (Supplemental Figures 3E–G).

Our data do show, in contrast, that Fen treatments mitigate the TLR4 and TNF-α inflammatory axis in the gut (Supplementary Figures S2). These findings could provide insight into Fen’s preservation of the gut barrier with alcohol intake, as mice lacking TLR4 or TNF-α receptor I (TNFRI) are protected against alcohol-mediated gut barrier dysfunction and endotoxemia (Uesugi et al., 2001; Chen et al., 2015). As retinoids are known to regulate differentiation of gut epithelial cells (Gudas, 2011), future studies using intestinal specific RAR null-mice to determine if Fen’s barrier preserving effect involves retinoid-mediated differentiation of intestinal epithelial cells are warranted.

### Fenretinide Inhibits Hepatic Steatosis and Liver Injury

Our data also show positive effects of Fen treatments on mitigating clinical hallmarks of ALD, including hepatic steatosis (Figures 2M,N) and liver injury (Figures 2O,P). The reductions in liver triglyceride content in EtOH + Fen-treated mice (Figure 3N) did not correlate with changes in hepatic expression of lipogenic genes such as FASN and SREBP1-c (Supplementary Figures 4A–D). As food intake and alcohol-exposure was unchanged between both EtOH experimental groups (Supplementary Figure 1B), it remains unclear how Fen reduces alcohol-mediated hepatic steatosis and liver injury. Moreover, we have evidence that the anti-ALD effects of Fen do not involve activation of RARs, as hepatic mRNA expression of the well-established retinoid-mediated target genes, RARβ2 and Cyp26A1 (Gudas, 2011), were unchanged in EtOH + Fen and pair-fed + Fen-treated mice compared to pair-fed and EtOH-treated mice respectively (Supplementary Figures 4E,F). This is consistent with the data showing that the anti-cancer actions of Fen are largely through non-RAR mediated pathways (Sheikh et al., 1995; Hail et al., 2006). Still, it is likely that Fen has unappreciated effects on the complex regulation of alcohol-mediated lipogenesis and lipid metabolism. Therefore, in future studies next-generation RNA sequencing could be performed to explore the hepatic gene networks impacted by Fen in chronic alcohol exposure.

### Fenretinide Reduces Hepatic Expression of Toll-like Receptor 4 and TNF-α

The concomitant reductions in endotoxemia (Figure 1B) and hepatic and Kupffer cell expression of TLR4 (Supplemental Figure S5) and TLR4 targets, including TNF-α (Figures 3I–L, 3U), in the livers of EtOH + Fen-treated mice could provide mechanistic insights into Fen’s mitigation of hepatic steatosis and liver injury, as LPS is a potent stimulator of the TLR4 inflammatory axis (Uematsu and Akira, 2006). Moreover, there is a convincing body of evidence of a nexus between the innate immune inflammatory axis and nutrient and metabolic pathways (Hotamisligil, 2006), including studies showing that TNF-α and LPS:TLR4 signaling promote lipogenesis in hepatocytes *in vivo* (Feingold and Grunfeld, 1987; Feingold et al., 1992; Jia et al., 2018). Nevertheless, we recognize that the preservation of gut barrier function (Figure 1A) and reductions in endotoxemia (Figure 1B) may not be mechanistically related to the reductions in hepatic steatosis (Figure 3M,N) and liver injury (Figures 3O,P), in EtOH + Fen-treated mice. Moreover, it is unclear if the modulations of TLR4 and TNF-α in both the guts and livers of EtOH + Fen-treated mice are a direct effect of Fen on TLR4 signaling, are from reductions in systemic endotoxemia, or both.

Future studies using hepatocyte and macrophage-specific TLR4 or TNFR1 mutant mice are warranted, and could provide a better understanding of the molecular pathways involved in Fen’s gut preserving and anti-ALD effects. Still, taken together, our data are consistent with a number of recent studies reporting that Fen possess anti-TLR4, anti-inflammatory, and TJP-preserving actions (López-Vales et al., 2010; Lachance et al., 2013; Kanagaratham et al., 2014; Lin et al., 2016).

## Conclusion

Given that few therapeutics for ALD have been developed which can preserve both gut integrity and liver functions, the data presented here suggest that Fen may possess properties that target both gut barrier function and clinical hallmarks of ALD. Also, given the decades of safety data for Fen from numerous human clinical trials (Veronesi et al., 2006), our findings warrant further pre-clinical testing of Fen as an anti-ALD drug.

## Data Availability

The raw data supporting the conclusions of this article will be made available by the authors, without undue reservation.
