# Supplementary material for: Fenretinide Improves Intestinal Barrier Function and Mitigates Alcohol Liver Disease
Source: Front Pharmacol. 2021 Mar 18;12:630557. doi: 10.3389/fphar.2021.630557 (PMC8012525; doi:10.3389/fphar.2021.630557)
Supplement: Supplementary file 1 [file table1.pdf]

Gene Name	Forward Primer (5'-3')	Reverse Primer (5'-3')
**Pathway: Tight Junction Proteins**		
Claudin 1	TGGGTTTCATCCTGGCTTCT	TGTATCTGCCCGGTGCTTT
Occludin	GCAGCCTTCTGCTTCATCG	CGTCGGGTTCACTCCCATTA
Zona-Occludins 1 (ZO-1)	TCACGATCTCCTGACCAACG	GGCTGACGGGTAAATCCACA
Cingulin	AGCAGAGCAGCAAGGAACTT	GCTGTAGCTCCTTCACCTGG
Fodrin/Spectrin alpha	CGCATCTTTTTCCTCAGCAG	CCAGGACTTGCTGTCGTCTC
Symplekin	CGGAGTGTGGCATCACAGTTT	CGCACTTCAATGGATTTGTCTG
**Pathway: Inflammation**		
Interleukin-1ß (IL1ß)	GAAATGCCACCTTTTGACAGTG	CTGGATGCTCTCATCAGGACA
Interleukin-6 (IL-6)	TAGTCCTTCCTACCCCAATTTCC	TTGGTCCTTAGCCACTCCTTC
Toll-like receptor 4 (TLR4)	ATGGCATGGCTTACACCACC	GAGGCCAATTTTGTCTCCACA
Tumor necrosis factor receptor 1(TNFR1)	CCGGGAGAAGAGGGATAGCTT	TCGGACAGTCACTCACCAAGT
Tumor necrosis factor-α (TNF-α)	CCTGTAGCCCACGTCGTAG	GGGAGTAGACAAGGTACAACCC
**Pathway: Oxidative Stress**		
Glutathione S-transferase, alpha 1(Gsta1)	AGCCCGTGCTTCACTACTTC	TCTTCAAACTCCACCCCTGC
Glutathione S-transferase, m1 (Gstm1)	GAGGGCCTCAAGAAGATCTCTG	TTACTCCAGTGGGCCATCTTTG
**Pathway: Retinoid Signaling**		
Retinoic acid receptor β (isoform 2) (RARβ2)	GCGAGAGTTTGATGGAGTTC	TGGTAGCCCGAGACTTGTCCT
Cytochrome P450 26A1 (CYP26A1)	GAAACATTGCAGATGGTGCTTCAG	CGGCTGAAGGCCTGCATAATCAC
**Pathway: Lipid Metabolism**		
Acetyl-Coenzyme A carboxylase alpha (ACC1)	ATGGGCGGAATGGTCTCTTTC	TGGGGACCTTGTCTTCATCAT
Fatty Acid Synthase (FASN)	GGAGGTGGTGATAGCCGGTAT	TGGGTAATCCATAGAGCCCAG
Peroxisome proliferator-activated receptor γ (PPARγ)	CTCCAAGAATACCAAAGTGCGA	GCCTGATGCTTTATCCCCACA
Sterol regulatory element binding transcription factor 1-c (SREBP1-c)	CGGAAGCTGTCGGGGTAG	GTTGTTGATGAGCTGGAGCA
**Pathway: Housekeeping**		
36B4	AGAACAACCCAGCTCTGGAGAAA	ACACCCTCCAGAAAGCGAGAGT
**Pathway: Universal Bacterial Primers**		
Universal bacterial 16s rRNA (J Clin Microbio 2005, 43:5332-7)	GTGSTGCAYGGYTGTCGTCA	ACGTCRTCCMCACCTTCCTC
