# Supplementary material for: Fenretinide Improves Intestinal Barrier Function and Mitigates Alcohol Liver Disease
Source: Front Pharmacol. 2021 Mar 18;12:630557. doi: 10.3389/fphar.2021.630557 (PMC8012525; doi:10.3389/fphar.2021.630557)
Supplement: Supplementary file 2 [file datasheet1.pdf]

Supplementary Material

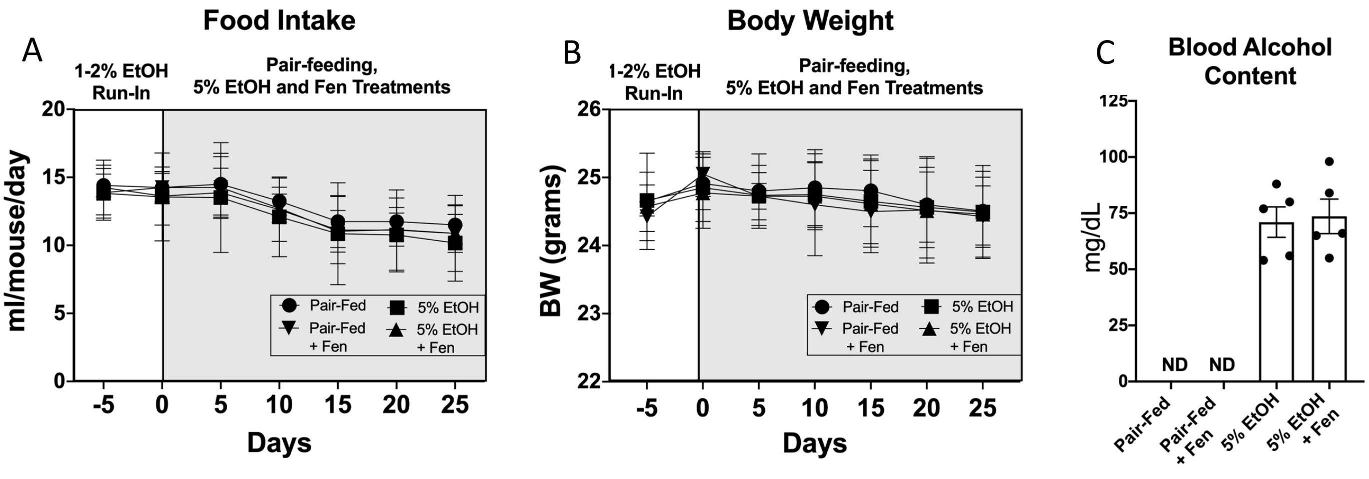

Supplementary Figure 1. A) Food intake in pair-fed (no EtOH) and 5% ethanol (EtOH)-fed C57BL/6 mice treated with either vehicle (0.1% DMSO) [pair-fed, EtOH-untreated] or fenretinide (10mg/kg/bw/day) [pair-fed-Fen, EtOH+Fen for 25 days. B) Body weight of mice described in A). C) Blood alcohol content (BAC) in mice 2 hours after peak blood alcohol content relative to pair-fed mice described in A). All data errors bars represent ± SD, with ND=not detected.

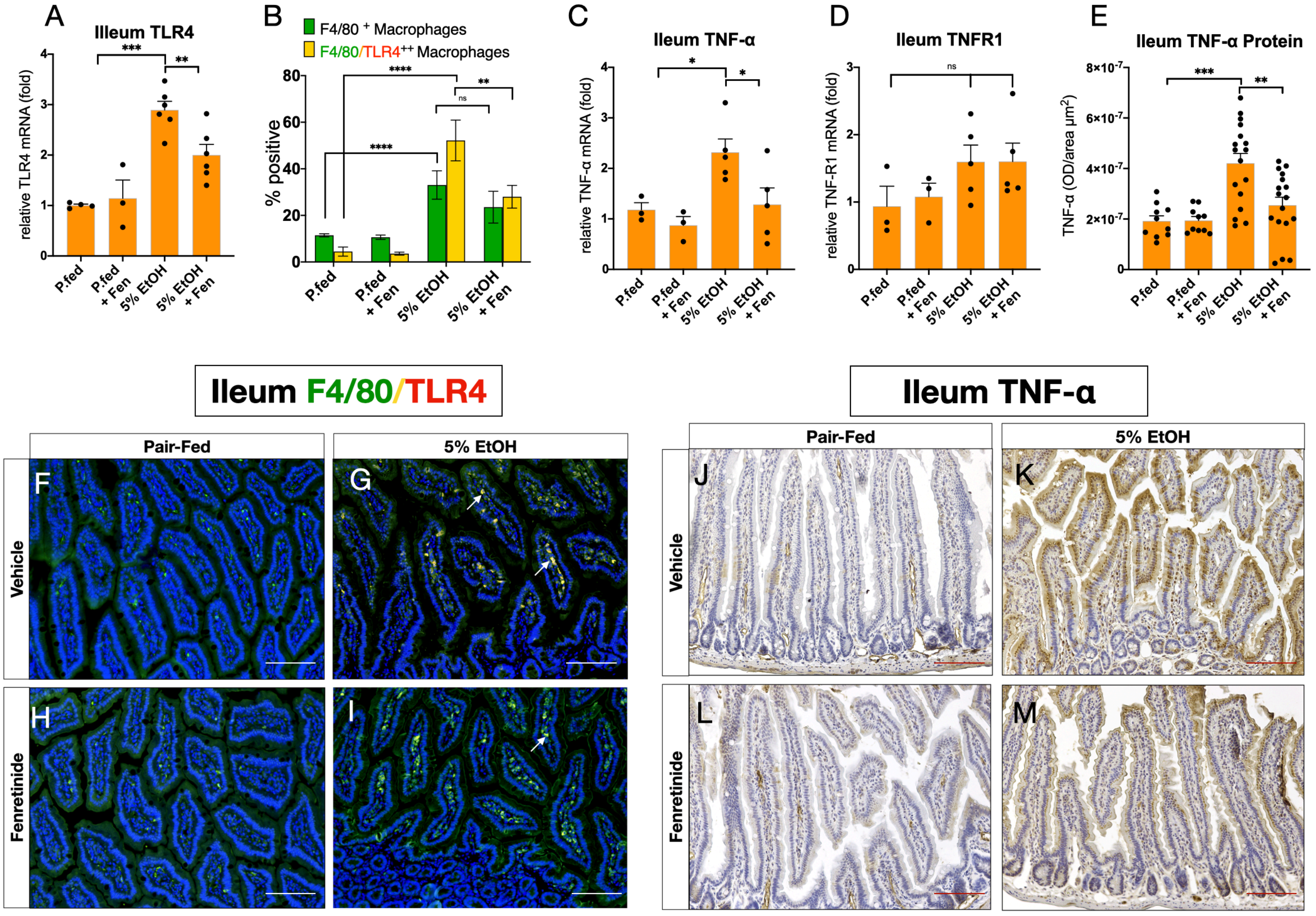

Supplementary Figure 2. A) Ileum mRNA levels of *TLR4,* B) Quantification of ileum immunofluorescence (IF) optical density of F4/80 and F4/80:TLR4 double positive macrophages. Ileum mRNA levels of C) *TNF-α, and* D*) tumor necrosis factor receptor 1 (TNFR1).* E) Quantification of ileum immunohistochemistry (IHC) for TNF-α protein levels. F-I) Representative double immunofluorescence (IF) images of ileum sections stained with antibodies against F4/80 (green channel) and TLR4 (red channel) with double positive cells (yellow/orange [white arrows]) Magnification: 100X; Scale Bar=50 µm. J-M) Representative immunohistochemistry (IHC) images of ileum sections stained with an antibody against tumor necrosis factor α (TNF-α) Magnification: 100X; Scale Bar=50 µm. All data errors bars represent ± SD, with *p<0.05, **p<0.01, ***p<0.001, ****p<0.0001, ns=not significant.

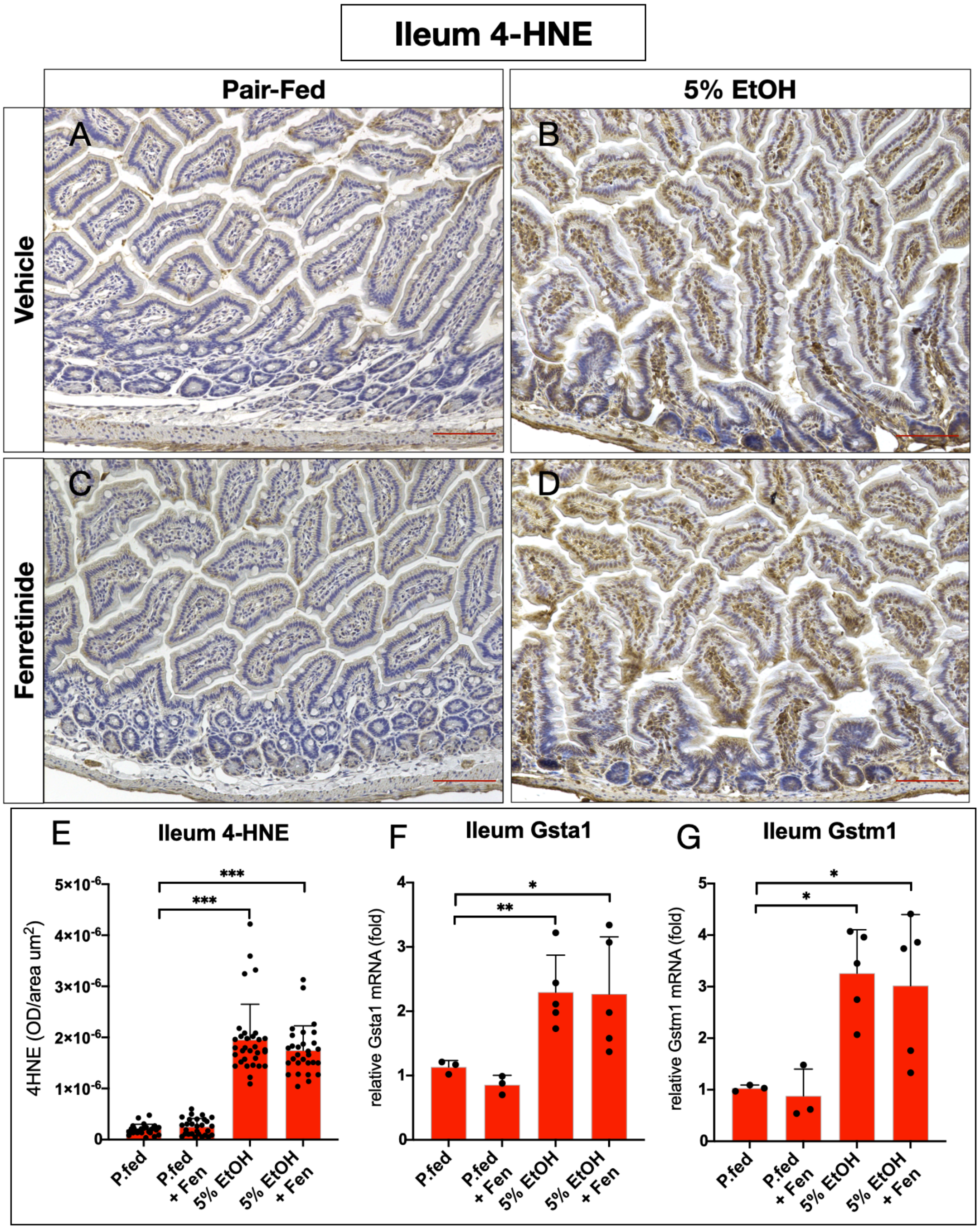

Supplementary Figure 3. A-D) Representative immunohistochemistry (IHC) images of ileum sections stained with an antibody against 4 hydroxynonenal (4-HNE) Magnification: 100X; Scale Bar=50 µm. E) Quantification of ileum IHC for 4-HNE protein levels. Ileum mRNA levels of Nrf-2 target genes F) Gsta1, and B), Gstm1. All data errors bars represent ± SD, with *p<0.05, **p<0.01, ***p<0.001, ****p<0.0001.

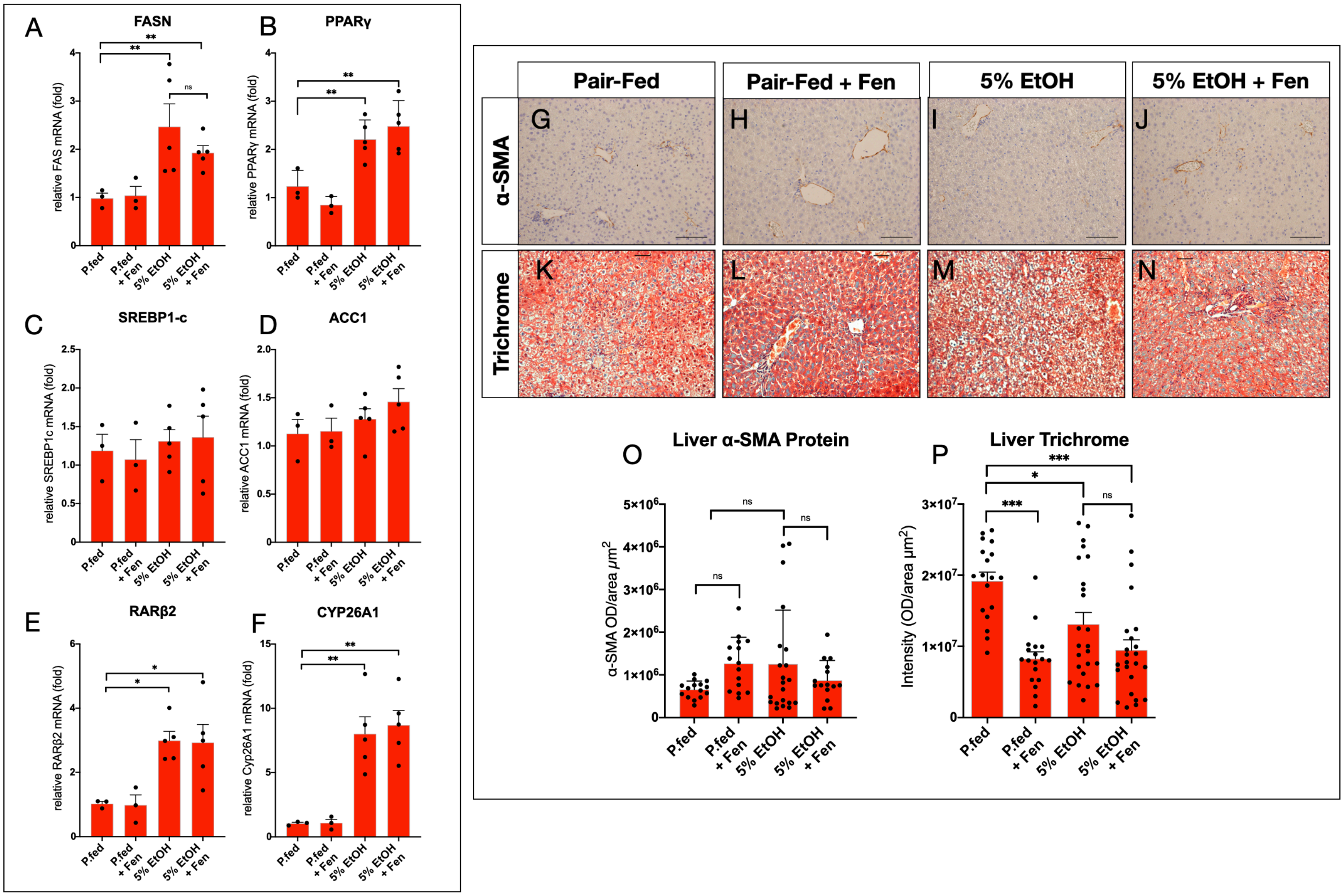

Supplementary Figure 4. Hepatic mRNA levels of lipogenic genes A) *FASN*, B) *PPAR-γ*, C) *SREBP1-c,* D) *ACC1* and retinoid target genes, E) *RARβ2*, and F) *Cyp26a1*.G-J) Representative immunohistochemistry (IHC) images of liver sections stained with an antibody against smooth muscle actin α (α-SMA) Magnification: 100X; Scale Bar=50 μm. K-N) Representative images of liver sections stained with Masson's trichrome for collagen. Magnification: 100X; Scale Bar=50 μm. Quantification liver M) IHC for α-SMA protein levels and N) Masson's trichrome for collagen. All data errors bars represent ± SD, with *p<0.05, **p<0.01, ***p<0.001, ns=not significant.

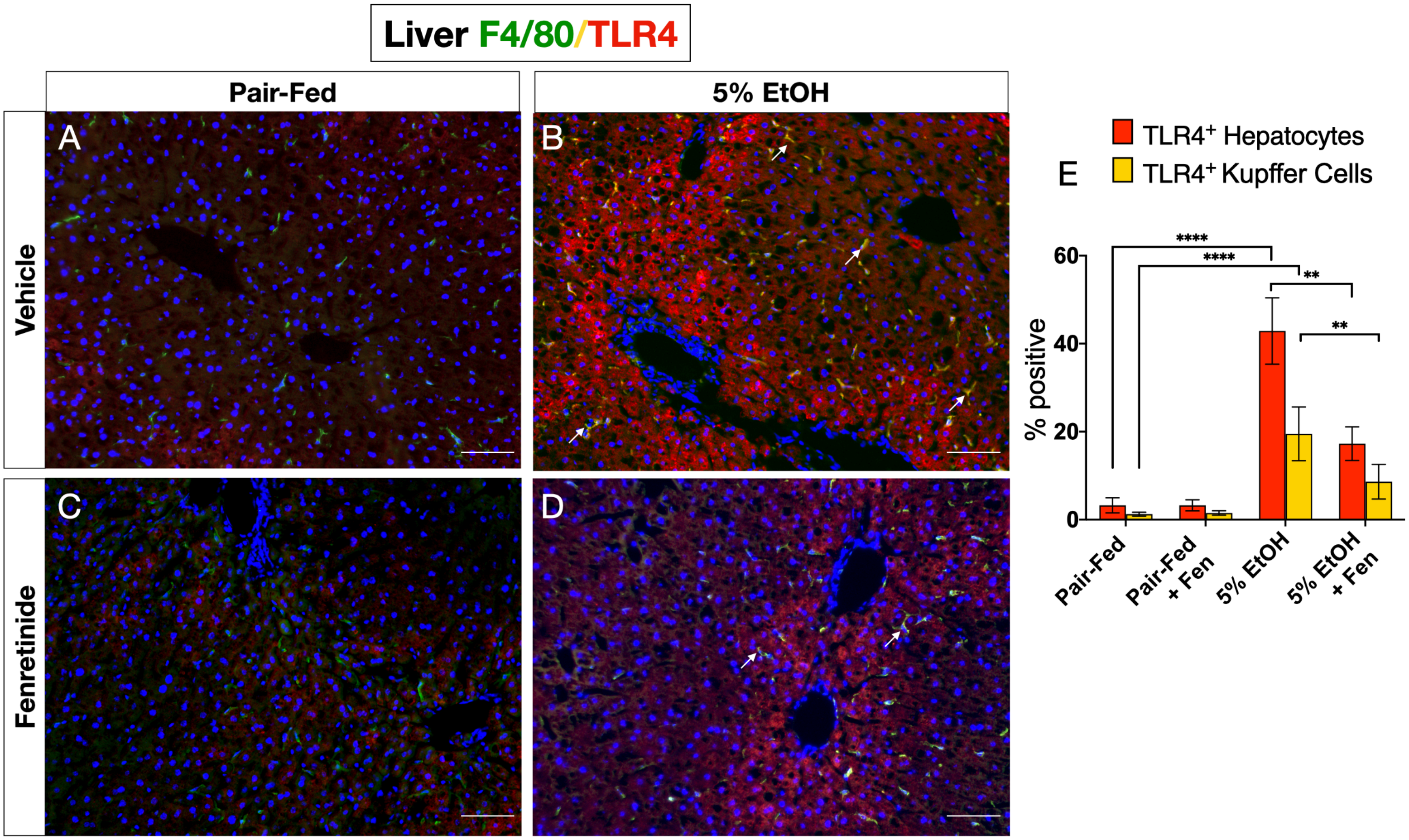

Supplementary Figure 5. A-D) Representative double immunofluorescence (IF) images of liver sections stained with antibodies against F4/80 (green channel) and TLR4 (red channel) with double positive cells (yellow/orange [white arrows]) Magnification: 100X; Scale Bar=50 µm. Quantification of liver immunofluorescence (IF) optical density of F4/80 and F4/80:TLR4 double positive macrophages. All data errors bars represent ± SD, with **p<0.01, ****p<0.0001.

Gene Name	Forward Primer (5'-3')	Reverse Primer (5'-3')
**Pathway: Tight Junction Proteins**		
Claudin 1	TGGGTTTCATCCTGGCTTCT	TGTATCTGCCCGGTGCTTT
Occludin	GCAGCCTTCTGCTTCATCG	CGTCGGGTTCACTCCCATTA
Zona-Occludins 1 (ZO-1)	TCACGATCTCCTGACCAACG	GGCTGACGGGTAAATCCACA
Cingulin	AGCAGAGCAGCAAGGAACTT	GCTGTAGCTCCTTCACCTGG
Fodrin/Spectrin alpha	CGCATCTTTTTCCTCAGCAG	CCAGGACTTGCTGTCGTCTC
Symplekin	CGGAGTGTGGCATCACAGTTT	CGCACTTCAATGGATTTGTCTG
**Pathway: Inflammation**		
Interleukin-1ß (IL1ß)	GAAATGCCACCTTTTGACAGTG	CTGGATGCTCTCATCAGGACA
Interleukin-6 (IL-6)	TAGTCCTTCCTACCCCAATTTCC	TTGGTCCTTAGCCACTCCTTC
Toll-like receptor 4 (TLR4)	ATGGCATGGCTTACACCACC	GAGGCCAATTTTGTCTCCACA
Tumor necrosis factor receptor 1(TNFR1)	CCGGGAGAAGAGGGATAGCTT	TCGGACAGTCACTCACCAAGT
Tumor necrosis factor-α (TNF-α)	CCTGTAGCCCACGTCGTAG	GGGAGTAGACAAGGTACAACCC
**Pathway: Oxidative Stress**		
Glutathione S-transferase, alpha 1(Gsta1)	AGCCCGTGCTTCACTACTTC	TCTTCAAACTCCACCCCTGC
Glutathione S-transferase, m1 (Gstm1)	GAGGGCCTCAAGAAGATCTCTG	TTACTCCAGTGGGCCATCTTTG
**Pathway: Retinoid Signaling**		
Retinoic acid receptor β (isoform 2) (RARβ2)	GCGAGAGTTTGATGGAGTTC	TGGTAGCCCGAGACTTGTCCT
Cytochrome P450 26A1 (CYP26A1)	GAAACATTGCAGATGGTGCTTCAG	CGGCTGAAGGCCTGCATAATCAC
**Pathway: Lipid Metabolism**		
Acetyl-Coenzyme A carboxylase alpha (ACC1)	ATGGGCGGAATGGTCTCTTTC	TGGGGACCTTGTCTTCATCAT
Fatty Acid Synthase (FASN)	GGAGGTGGTGATAGCCGGTAT	TGGGTAATCCATAGAGCCCAG
Peroxisome proliferator-activated receptor γ (PPARγ)	CTCCAAGAATACCAAAGTGCGA	GCCTGATGCTTTATCCCCACA
Sterol regulatory element binding transcription factor 1-c (SREBP1-c)	CGGAAGCTGTCGGGGTAG	GTTGTTGATGAGCTGGAGCA
**Pathway: Housekeeping**		
36B4	AGAACAACCCAGCTCTGGAGAAA	ACACCCTCCAGAAAGCGAGAGT
